# Comparison of choriocapillary flow density between fellow eyes of polypoidal choroidal vasculopathy and neovascular age-related macular degeneration

**DOI:** 10.1186/s12886-020-01386-0

**Published:** 2020-04-22

**Authors:** Mingyue Luo, Xinyu Zhao, Nan Zhao, Mingzhen Yuan, Jingyuan Yang, Rongping Dai, Youxin Chen

**Affiliations:** 1 Department of Ophthalmology, Peking Union Medical College Hospital, Chinese Academy of Medical Sciences, Beijing, 100730 China; 2 Key Lab of Ocular Fundus Disease, Chinese Academy of Medical Sciences, Beijing, 100730 China; 3 Central Laboratories, Peking Union Medical College Hospital, Chinese Academy of Medical Sciences, Beijing, 100730 China

## Abstract

**Background:**

To compare the choriocapillary flow density (CFD) among the fellow eyes of polypoidal choroidal vasculopathy (PCV), neovascular age-related macular degeneration (nAMD), and healthy controls using spectral-domain optical coherence angiography tomography (SD-OCTA).

**Methods:**

This is a cross-sectional study that includes the fellow eyes of 38 patients with unilateral PCV, 36 patients with unilateral nAMD, and 36 eyes from 36 healthy volunteers. The PCV group was further classified into polypoidal CNV (P-CNV) and typical PCV (T-PCV) for subgroup analysis. The age, subfoveal choroidal thickness (SFCT), Age-Related Eye Disease Study (AREDS) classification, and fellow eye diagnosis were acquired. All subjects underwent SD-OCTA with a 6.0-mm scan pattern. Circles with radius of 1.00, 1.50, and 3.00 mm were manually selected in the choriocapillaris (CC) slab, and the CFD was calculated as the percentage of the flow area to the whole selected area as CFD-1.00, 1.50, and 3.00, respectively. Univariate and multivariate analysis were performed to study the correlation between the aforementioned factors with CFD.

**Results:**

The mean CFD-1.00, 1.50, and 3.00 of the nAMD group were 61.51, 63.18, and 66.20, respectively; these were significantly lower than those of the PCV group (65.90, 66.89, and 67.94; *P* < 0.001, *P* < 0.001, and *P* = 0.010; respectively) and control group (66.28, 66.96, and 68.42; *P* < 0.001, *P* < 0.001, and *P* = 0.001, respectively), and no difference was detected between the PCV and control group or between PCV subtypes. The AREDS classification and fellow eye diagnosis were correlated with CFD in univariate analysis; however, only the fellow eye diagnosis showed a significant correlation after multiple linear regression.

**Conclusions:**

The CFD of nAMD fellow eyes was significantly lower than that of PCV and control eyes, and no difference was detected between PCV and control group, indicating that CC loss plays a different role in the early pathogenesis of nAMD and PCV.

## Background

Polypoidal choroidal vasculopathy (PCV) is characterized by orange-red nodules in fundus examinations, polypoidal lesions during indocyanine green angiography (ICGA), and tremendous bleeding at the posterior pole. Since it was first described by Yannuzi in the 1980s [1], there has been many controversies, especially the pathogenesis and whether PCV is a subtype of neovascular age-related macular degeneration (nAMD), or a distinct disease within the pachychoroid disease spectrum [2], owing to the obvious heterogeneity that lies in their clinical, pathophysiological and epidemiological features and treatment responses to anti-vascular endothelium growth factor (VEGF) agents [3].

The pathogenesis and nature of PCV as well as its characteristic lesions, namely polyps and the branching vascular network (BVN), remain unclear. In recent years, multimodal imaging technologies have allowed more precise views of polypoidal lesions and PCV choroids. Specifically, with optical coherence tomography angiography (OCTA), a novel imaging modality that reflects the choroid flow with higher accuracy and resolution compared to traditional dye-based ophthalmic angiography [4], emerging evidence suggests that the classical polypoidal lesions manifest as tangled vessels at the edge of BVN [5]. This finding further complicates our understanding of whether there is an actual difference between PCV and nAMD because the characteristic lesions of PCV and choroidal neovascularization (CNV) in nAMD are essentially both vascularization in nature and there might actually be no “polyps” or “aneurysmal vessels” [6].

Despite various rates of bilateral presentation [3], many studies have identified asymptomatic lesions in fellow eyes of both PCV and nAMD patients. For example, nonexudative neovascularization of the fellow eye was found to be associated with pachychoroid pigment epitheliopathy in PCV and nAMD patients [7], and an increased trend of choriocapillaris (CC) nonperfusion was detected in the fellow eyes of nAMD using OCTA [8]. Additionally, retinal pigmented epithelium (RPE) and outer retinal abnormalities were observed in 84% of a cohort of unilateral PCV patients, and CNV formation was observed more if these abnormalities were accompanied with pachyvessels [9]. These studies, among other fellow eye studies, may shed light on the pathogenesis, especially in the early phases. It remains unclear whether there is a direct causal effect between pachychoroid and the pathogenesis of PCV, yet RPE alterations seem to be a secondary downstream event occurring in the later stage of the disease. This differs from nAMD, in which the interplay between RPE and CC [10, 11] plays a central role in the early phase, and finally, elevated VEGF leads to CNV.

CC flow density (CFD) detected using OCTA have emerged as a potential metric to evaluate the inner choroid vasculature through fast noninvasive imaging and have therefore been used extensively for various retinal diseases [12–15]. However, PCV and nAMD in their fellow eyes have not been compared using OCTA. This study aimed to compare the fellow eyes of PCV and AMD in terms of CFD at an early stage without signs of neovascularization.

## Methods

This is a cross-sectional, observational study of unilateral PCV and nAMD patients recruited from June 2017 to July 2019 at Peking Union Medical College Hospital. Healthy controls were also included. This study was approved by the institutional review board (S-K631) and was conducted in accordance with the tenets of the Declaration of Helsinki. Written informed consent was waived because of the study’s retrospective design.

PCV and nAMD were diagnosed according to published criteria [1, 16]. Two masked ophthalmologists (M.L. and M.Y.) reviewed the early to late phases of the angiography images, verified the diagnosis, and classified PCV cases into polypoidal CNV (P-CNV) or typical PCV (T-PCV) [17]. A third retinal consultant (Y.C.) arbitrated in case of any discrepancy. The inclusion criteria for patients were unilateral PCV or nAMD cases with complete medical records. Patients and controls were excluded if (1) any signs of neovascularization in the included eyes, namely, exudation in FFA or ICGA for patients and BVN or blood flow signals between RPE and Bruch’s membrane in OCTA for all; (2) comorbid with other ophthalmic diseases except for myopia less than − 3.0D and mild cataract; (3) cloudy refractory media impedes examination of the posterior pole; and (4) OCTA scan quality was lower than 7.

All patients received comprehensive ophthalmologic examinations, including Snellen best-corrected visual acuity (BCVA), SD-OCTA (RTVue XR Avanti AngioVue; Optovue, Inc., Fremont, CA, USA), simultaneous fluorescein angiography ICGA, and enhanced depth imaging (EDI) SD-OCT (Heidelberg Retina Angiography 2, HRA2, Heidelberg Engineering, Heidelberg, Germany). For statistical analysis, the Snellen BCVA was converted to a logarithm of the minimal angle of resolution (logMAR).

All included eyes were stratified according to Age-Related Eye Disease Study (AREDS) classification [18], (1) No apparent aging changes or normal aging changes; (2) Early AMD; (3) Intermediate AMD; and (4) Late AMD, based on drusen size and pigmentary abnormalities (AREDS category 1–4, respectively). Subfoveal choroidal thickness (SFCT) was measured using EDI-OCT horizontal B-scans as the distance between the Bruch membrane and the choroid-scleral border. An HD Angio Retina 6.0-mm scan pattern of each subject was acquired and used for further analysis. Motion correction technology and 3D projection artifact reduction were used automatically. Circles centered at the fovea with radius of 1.00, 1.50, and 3.00 mm were manually adjusted in the CC slab using built-in software, and the CFD was calculated as the percentage of flow area to the whole selected area and generated automatically as CFD-1.00, 1.50, and 3.00, respectively.

### Statistical analysis

Statistical analyses were performed using SPSS 22 (IBM, Inc., Chicago, IL). Normally distributed continuous data were presented as the mean and standard deviation (SD) or as the median and interquartile range (IQR) in the case of abnormal distribution. A *P* value less than 0.05 was considered significant in all aforementioned analysis. Categorical and continuous variables were assessed using a chi-square test and one-way analysis of variance (ANOVA), respectively, with post-hoc Bonferroni correction. Bivariate relationships were assessed using Spearman correlation coefficient analysis to evaluate the potential correlating factors of CFD. Multivariate linear regression analysis was performed with age, SFCT, and other factors with the significance from univariate analysis as dependent factors.

## Results

The fellow eyes of 38 patients with unilateral PCV (mean age: 63.8 ± 8.8 years), 36 patients with unilateral nAMD (mean age: 67.1 ± 8.4 years), and 36 eyes from 36 healthy volunteers (mean age: 63.4 ± 7.1 years) were included in this study. The subjects were matched in terms of age, gender, BCVA, smoking status, hypertension, and diabetes mellitus (DM) among the groups. Table 1 summarizes the basic demographic and clinical characteristics of the subjects.

**Table 1:** Baseline demographics and clinical characteristics of subjects

······	PCV (*N* = 38)	nAMD (*N* = 36)	Control (*N* = 36)	*P* value (PCV vs. AMD)	*P* value (PCV vs. control)	*P* value (AMD vs. control)
Age, y mean (SD)	63.8 (8.8)	67.1 (8.4)	63.4 (7.1)	0.270	1.000	0.171
Gender, female (N) (%)	20 (52.6)	12 (33.3)	22 (61.1)	0.282	1.000	0.054
BCVA (logMAR) (IQR)	0.09 (0.00–0.10)	0.12 (0.00–0.22)	0.08 (0.00–0.10)	0.676 ^a^	0.942 ^a^	0.344 ^a^
Smoker/non-smoker	4/34	5/31	3/33	1.000 ^b^	1.000 ^b^	1.000 ^b^
Hypertention (%)	4 (10.5)	9 (25.0)	5 (13.9%)	0.306	1.000	0.702
DM (%)	4 (10.5)	4 (11.1)	1 (2.8)	1.000 ^b^	0.507 ^b^	0.453 ^b^
AREDS Category 1/2/3/4	34/3/1/0	21/7/8/0	36/0/0/0	0.012 ^b^*	0.186 ^b^	0.001^b^*
SFCT (μm)	242.97 (53.59)	196.61 (45.58)	209.94 (46.22)	< 0.001*	0.013*	0.744
CFD-1.00 (%)	65.90 (3.47)	61.51 (3.90)	66.28 (3.39)	< 0.001*	1.000	< 0.001*
CFD-1.50 (%)	66.89 (3.30)	63.18 (3.42)	66.96 (2.96)	< 0.001*	1.000	< 0.001*
CFD-3.00 (%)	67.94 (2.52)	66.02 (3.10)	68.42 (2.59)	0.010*	1.000	0.001*

*SD* Standard deviation, *BCVA* Best-corrected visual acuity, *logMAR* Logarithm of the minimal angle of resolution, *IQR* Interquartile range, *DM* Diabetes mellitus, *AREDS* Age-Related Eye Disease Study, *SFCT* Subfoveal choroidal thickness, *CFD* Choriocapillaris flow deficit, *PCV* Polypoidal choroidal vasculopathy, *nAMD* Neovascular age-related macular degeneration

*Statistically significant *P* value. ^a^ Post-hoc Tambane correction. ^b^ Fischer’s exact test

The AREDS category of the AMD group was more advanced compared to those of the PCV and control group (*P* = 0.012 and 0.001, respectively), with seven and eight subjects classified as AREDS category 2 and 3, respectively. SFCT of the PCV group was significantly greater than those of the AMD and control group (242.97 ± 53.59 μm vs 196.61 ± 45.58 μm and 209.94 ± 46.22 μm, *P* < 0.001 and *P* = 0.013, respectively). Mean CFD-1.00, 1.50, and 3.00 (SDs) of the nAMD group were 61.51, 63.18, and 66.20, respectively; these were significantly lower than those of the PCV group (65.90, 66.89, and 67.94; *P* < 0.001, *P* < 0.001, and *P* = 0.010, respectively) and control (66.28, 66.96, and 68.42; *P* < 0.001, *P* < 0.001, and *P* = 0.001, respectively), and no difference was detected between the PCV group and the control.

Univariate analysis (Table 2) indicated that CFD-1.00, CFD-1.50, and CFD-3.00 were all correlated with the AREDS classification (*P* = 0.001, 0.006, and 0.049, respectively) and fellow eye diagnosis (*P* < 0.001, *P* < 0.001, and *P* = 0.001, respectively). Multiple linear regression (Table 3) revealed that in this study cohort, fellow eye diagnosis was the only factor strongly correlated with CFD (*P* < 0.001, *P* < 0.001, and *P* = 0.001, respectively).

**Table 2:** Univariate analysis of potential correlation factors (Age, gender, BCVA, smoking status, hypertension, DM, AREDS classification, and SFCT) With CFD-1.00, CFD-1.50, and CFD-3.00 as dependent variable

Characteristic	CFD-1.00		CFD-1.50		CFD-3.00	
	Correlation Coefficient	*P* value ^a^	Correlation Coefficient	*P* value ^a^	Correlation Coefficient	*P* value ^a^
Age	−0.06	0.537	0.01	0.921	−0.05	0.617
Gender	−0.07	0.483	−0.06	0.563	−0.13	0.895
BCVA	−0.01	0.902	0.03	0.750	0.05	0.581
Smoking status	−0.08	0.388	−0.07	0.478	−0.09	0.340
Hypertension	−0.07	0.453	−0.063	0.515	< 0.01	0.974
DM	−0.08	0.428	−0.06	0.519	−0.12	0.221
AREDS classification	−0.30	0.001*	−0.26	0.006*	−0.19	0.049*
SFCT	0.11	0.244	0.10	0.284	0.06	0.520
Fellow eye diagnosis	−0.46	< 0.001*	−0.42	< 0.001*	− 0.32	0.001*

*DM* Diabetes mellitus, *AREDS* Age-Related Eye Disease Study, *SFCT* Subfoveal choroidal thickness, *CFD* Choriocapillaris flow deficit

*Statistically significant *P* value. ^a^ Spearman correlation analysis

**Table 3:** Multiple linear regression analysis of correlation factors with CFD-1.00, CFD-1.50 and CFD-3.00 as dependent variables

	CFD-1.00		CFD-1.50		CFD-3.00	
	β	*P* value	β	*P* value	β	*P* value
Fellow eye diagnosis	−0.443	< 0.001*	− 0.437	< 0.001*	−0.334	0.002*
AREDS Classification	−0.166	0.082	−0.399	0.691	−0.059	0.572
Age	0.059	0.518	0.109	0.249	−0.007	0.947
SFCT	−0.038	0.671	−0.037	0.691	−0.055	0.576

*AREDS* Age-Related Eye Disease Study, *SFCT* Subfoveal choroidal thickness, *CFD* Choriocapillaris flow deficit

*Statistically significant *P* value

There were 13 P-CNV and 25 T-PCV patients in the PCV group. SFCT of the T-PCV group was significantly greater than those of the P-CNV, nAMD and control group (265.08 ± 43.68 μm vs 200.46 ± 45.44 μm, 196.61 ± 45.58 μm and 209.94 ± 46.22 μm, all *P* < 0.001). CFD-1.00 and CFD-1.50 of the P-CNV and T-PCV groups were significantly higher than that of the nAMD group but not significantly different from that of the control group (Supplementary file, Table 4). CFD-3.00 of the nAMD group was significantly lower than those of the T-PCV and control group but not significantly different from that of the P-CNV group. All CFD values were not significantly different between PCV subtypes (Supplementary file, Table 4). The univariate analysis and multiple linear regression results were similar. (Supplementary file, Tables 5 and 6).

## Discussion

In this study, we evaluated the CFD of fellow eyes of nAMD and PCV groups using OCTA and found that fellow eyes of the nAMD group had significantly lower CFD compared with the PCV and control group, and the differences between the PCV group and the control group or between PCV subtypes were not statistically significant. Although CFD was found to be negatively correlated with aging [19], it is unlikely that aging itself can account for the difference because the PCV, AMD, and control group were age-matched. Another important factor is the existence of drusen, which is considered a hallmark of dry AMD. In this study, all included eyes were stratified according to the AREDS classification, and more subjects with advanced categories were observed in the nAMD group. However, this index was not statistically significant in multivariate linear regression analysis. Although there is considerable overlap between the PCV and the nAMD groups, the difference in the CFD of their fellow eyes may serve as evidence of their heterogeneity.

The interplay of RPE and CC loss has always been of interest in the pathogenesis of AMD [20–23], although the sequential order remains debatable. Indeed, PCV is a well-recognized disease within the pachychoroid disease spectrum, and it is characterized by the dilation of the Haller layer and the attenuation of choriocapillaires and the Sattler layer. The engorgement of the vortex vein is often observed in PCV, with correlating choroidal hyperpermeability [24], as also confirmed by the pathology showing atherosclerotic changes in the choroid vessel wall and massive exudation of fibrin and blood plasma at polypoidal lesions [25, 26]. Apoptosis of smooth muscle cells and choroidal endothelial cells was also observed [25]. Chen et al. induced polyp-like structures by ligating vortex veins in cynomolgus monkeys [27]. These findings suggest the role of choroidal hemodynamics in PCV pathogenesis [28]. The CFD of fellow eyes of the PCV group was not significantly different from that of the control group, indicating that RPE-Bruch’s membrane-CC complex may not be involved in the preclinical stage of PCV and may be a downstream effect secondary to the primary pathology. Specifically, the near-normal CFD of fellow eyes of the PCV group does not contradict the vital role of CC flow deficits and RPE changes in pachychoroid pathogenesis, which may be a downstream event secondary to the dilation of the Haller layer. Thus, in the relatively healthy contralateral eyes of PCV, CFD may be near-normal and may possibly be accompanied with some pachychoroid features. Alternatively, this might be associated with the lower bilaterality of PCV eyes. Further studies regarding PCV laterality and the sequential order of choroidal changes in PCV are necessary for elaboration.

Many studies have investigated the difference in choroidal morphology between PCV and nAMD. SFCT is often used as an index; however, it fluctuates with the circadian rhythm and correlates with age and refractive errors [29]. Bakthavatsalam et al. [29] found that the choroidal vascular index (CVI) of an nAMD group was lower than that of a PCV group (64.94 vs 62.54); however, the difference was not statistically significant (*P* = 0.10). Pachyvessels, especially a diffuse pattern, were observed more commonly in thick-choroid PCV [30], and the vascular area of typical PCV was significantly larger than that of nAMD [31]. All of the abovementioned results indicate that the two diseases are heterogeneous in terms of choroidal morphology.

Our study had the inherent limitations of a retrospective cross-sectional study. Besides, its sample size is relatively small. Lastly, DM, hypertension, and smoking status were collected from patients’ self-reported past medical records; thus, they were not accurate enough to be included in the multivariate analysis. Nevertheless, to the best of our knowledge, this is the first study that has provided a quantitative analysis of CC among the fellow eyes of PCV, nAMD, and healthy eyes. Our study may provide evidence for the heterogeneity of nAMD and PCV.

## Conclusion

In conclusion, our findings suggest that CFD of nAMD fellow eyes was significantly lower than those of PCV and control eyes, and no difference was detected between PCV and control group or between PCV subgroups, indicating that CC loss plays a different role in the early pathogenesis of nAMD and PCV.

## Supplementary information

**Additional file 1: Table 4.** Baseline Demographics and Clinical Characteristics of Subjects. **Table 5.** Univariate Analysis of Potential Correlation Factors (Age, gender, BCVA, smoking status, hypertension, DM, AREDS classification, and SFCT) With CFD-1.00, CFD-1.50, and CFD-3.00 as dependent variable. **Table 6.** Multivariate Analysis of Correlation Factors with CFD-1.00, CFD-1.50 and CFD-3.00 as Dependent Variables.

## Data Availability

The datasets used and analyzed during the current study are available from the corresponding author upon reasonable request.

## Abbreviations

PCV: Polypoidal choroidal vasculopathy
ICGA: Indocyanine green angiography
nAMD: Neovascular age-related macular degeneration
VEGF: Vascular endothelium growth factor
BVN: Branching vascular network
OCTA: Optical coherence tomography angiography
CNV: Choroidal neovascularization
CC: Choriocapillaris
RPE: Retinal pigmented epithelium
CFD: CC flow density
P-CNV: Polypoidal CNV
T-PCV: Typical PCV
BCVA: Best-corrected visual acuity
EDI: Enhanced depth imaging
logMAR: Logarithm of the minimal angle of resolution
AREDS: Age-Related Eye Disease Study
SD: Standard deviation
IQR: Interquartile range
ANOVA: Analysis of variance
DM: Diabetes mellitus
